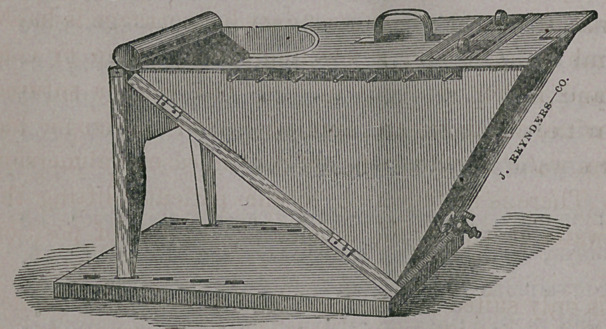# On the Use of Warm and Hot Water in Surgery

**Published:** 1876-02

**Authors:** Frederick E. Hyde

**Affiliations:** New York


### BUFFALO

### Medical and Surgical Journal.

VOL XV. FEBRUARY, 1876. No. 7.

### Original Communications.

:o:

ART. I.—*Warm and Hot Water in Surgery. A Short Historical Sketch, with the Present Most Approved Methods of Application. With Cases.* By FREDERICK E. HYDE, M. D., New York. [CONTINUED.]

CASE XVI.—*Large Bursa of Wrist—Incision and Submersion—Results negative.*—A blacksmith presented himself at my office with a large bursa on the back of his hand and forearm—probably seven inches in length by three in breadth, and by which he was completely disabled.

In February, 1874, I opened the bursa at two points, (at Bellevue Hospital,) and had the arm submerged at once in warm water. The openings did not prove to be sufficiently free to allow of easy ingress and egress of the water, and the consequence was that when suppuration took place the matter made an exit at two other points; but at no time was the inflammatory reaction violent.

March 25th.—The wounds are nearly closed, and it is apparent that a cure may be promised. In order to compare the results in this case with other similar cases, I will mention that about the same time a similar bursa of the arm was opened by one of my colleagues at Bellevue, and treated by injection of tinc. of iodine.

A very smart inflammatory reaction resulted, with a suppuration, and several openings formed for the discharge of pus. * * A few days since a patient was brought to me by Dr. Cox, of Harlem, with a similar bursa, which had opened spontaneously at various points, and was accompanied with purulent infiltrations between the metatarsal bones.

REMARKS.—On the whole, I am satisfied that of these three examples the case treated by submersion did the best, yet the value of this plan can only be demonstrated when the water has perfectly free ingress and egress. In a like case I would hereafter lay open the whole length of the bursa.

I may add here the following case of compound fracture of the first phalanx of the little finger, from my own practice.

R. S., a laborer, while carrying a stove, fell, a corner falling upon the little finger of the right hand, causing a compound, comminuted fracture at the middle of the phalanx, and a superficial wound of ring finger, the two wounds facing each other at the interdigital commissure.

Immediately after the accident he went to the nearest physician who placed it upon a straight wooden splint extending from the end of the finger to the wrist. This caused such excessive pain that he was unable to sleep the following night. Coming to me the day following, as I had previously attended his family, I removed the splint, found the the finger about half severed from the hand, with much contusion of margin of the wound. Directed the patient to put the hand in water as hot as he could comfortably bear it, and to keep it at such a temperature, using a deep bowl, and to cover the bowl with a cloth and oiled silk, at night to apply the warm, moist cloths and oiled silk. The submersion was continued about eight days.

The next day I found that the patient had rested well during the night.

Fourth day finger much swollen, about double normal size. Perceiving some odor from the wound, cut open the loosened, white and sodden epidermis, and found that the contused distal margin of the wound had sloughed and was partly separable, applied acetate of aluminum in dry powder to the wound, which removed the foul odor, leaving no odor whatever, as carbolic acid does. This was suggested by Dr. A. Rose. There was a decided line of demarcation between the healthy tissue and the gangrenous mass. The whole length of finger, from which the loosened epidermis had been removed, was of a bright pink, a new epidermis having already formed. The patient had experienced for the last forty-eight hours considerable pain from the swelling of the epidermis of the portion of the hand submerged, this was relieved by the addition of salt to the water. In a few days bright granulations had completely filled up the wound and new skin was advancing from the margins. Subsequently, noticing a delay in the healing process, I found an opening through the granulations leading to necrosed bone; being firmly wedged, I waited a few days for its spontaneous separation, but was finally obliged to remove it forcibly, taking out three pieces equal to the semi-circumference of the bone. Fibrous union had already taken place between the ends of the healthy bone. The wound now healed rapidly under fomentations. At no time did inflammation extend beyond the wound, or was pain felt in moving the tendons.

The œdema being very great I used no splint, as it was the little finger that was injured, and as the wound was on the side of, and close to, the interdigital commissure, I relied upon the contraction of the wound in cicatrizing to draw the finger into line. This it has done, and at the present writing, ten weeks after the accident, the finger has returned to its normal size, with wound completely healed. There is yet some passive anchylosis which would have been overcome before this, had not the patient ceased to manipulate it, having scraped the skin from the back of his finger in moving some furniture.

In a summary of conclusions drawn from these cases, Dr. Hamilton says: "No treatment hitherto adopted, under our observation, has been attended with equally favorable results. Under this plan the area of acute inflammation is exceedingly limited; erysipelatous inflammation has been almost uniformly arrested or restrained, when it has actually commenced, and it has never originated after submersion; gangrene has in no instance extended beyond the parts originally injured, and when progressing it has in most cases been speedily arrested, (in gangrene hot water, or water at a temperature of 100° to 110° Fahrenheit, is to be preferred). Septicaemia and pyaemia have not ensued in any case in which submersion has been practiced from the first day of the accident. Purulent infiltrations and consecutive abscesses have been infrequent, and always limited to the neighborhood of the parts injured, and small extent. Traumatic fever, usually present after grave accidents, when other plans of treatment have been pursued, as early as the third or fourth day, has seldom been present when this plan has been adopted, and in no case has the fever been intense or alarming."

"The phenomena usually observed in cases of recent lacerated or incised wounds, when submerged, are a sense of comfort, yet not absolute relief from pain; on the second and third day the parts adjacent are swollen, but not much reddened; the integument generally assumes a white and sodden appearance, and with only slight tenderness. On the fifth, sixth, or seventh day the swelling is greater than usually accompanies other plans of treatment; and, with the inexperienced, is likely to excite alarm, but it is found not to be attended with increased tenderness, and it pits upon pressure, showing that it is a condition of œdema only. At this time the granulations are generally covered with lymph or some exudate of a whitish color, and which might easily be mistaken for diphtheritic deposit. At the end of fourteen days, or thereabouts (the period at which, in most cases, we substitute fomentations for submersion), the limb is still œdematous, the granulations are abundant, sometimes presenting a fresh, red appearance, and at others covered with the white exudate."

### EFFECTS ENSUING FROM THE SUBSITUTION OF FOMENTATION FOR SUBMERSION.

"Pursuing the clinical history of these cases, we find that after fomentations are commenced, the œdema gradually lessens, but its final disappearance is delayed sometimes to a period beyond the complete cicatrization, so that the cicatrix, not unfrequently, is considerably depressed below the level of the sound parts; and we have seen this condition of the parts continue for many months.

We observe, also, that the granulations are red and abundant, and that cicatrization progresses as rapidly as under any other plan of treatment; indeed, we are inclined to think that it progresses more rapidly than we are accustomed to see where other plans, considered appropriate, have been adopted. We speak especially of the vigorous appearance of the granulations, and of the cicatrization, because our opinion was, before observing these cases, that warm water fomentations and warm baths would render granulations weak, pale, and sodden, and retard cicatrization. If such effects have resulted, they must have been presented as rare exceptions, since we have not observed them."

### AFTER ERYSIPELAS HAS ACTUALLY COMMENCED.

"We had very few oportunities of testing warm water submersion *after erysipelas has actually invaded a limb*, since, at St. Francis' Hospital, where most of these observations have been made, erysipelas is generally *prevented;* but in two or three examples imperfectly managed, at Bellevue Hospital, the results have at least furnished no testimony which would deter us from further trial. In explanation of this latter statement, relating to Bellevue Hospital, it is necessary to say that, with one exception, the bathing-tubs employed were improvised and were imperfect, the amount of attendance during the night is too limited to insure faithful attention to the temperature of the water, the heat of the wards is greatly lowered, and none of the nurses have experience in the management of the baths."

### VALUE OF HOT WATER IN TRAUMATIC GANGRENE.

"The power of hot water baths, or water at or above the normal temperature of the blood, to arrest traumatic gangrene is remarkable, and the writer entertains a hope that its efficiency may not be limited to *traumatic* gangrene alone, yet this remains to be proven."

In connection with the subject of erysipelas above referred to I have obtained the following five cases treated with warm water, from an article upon this subject, by Dr. A. H. Goeler, of New York, publishes in the *American Medical Weekly*, of Louisville, Ky., May 15, 1875.

Dr. Goeler was upon the staff at Ninety-ninth Street Reception Hospital at the time that Dr. Hamilton was surgeon-in-chief. After stating the advantages of the warm water treatment, over the remedies in general use for combatting traumatic erysipelas, such as cold and astringent applications, and painting the parts with tincture of iodine; he gives the cases, which where treated by himself, as follows:

Case I.—William Dunham, aged thirteen, native of the United States, was admitted February 29, 1874, with a compound comminuted fracture of lower end of the humerus and laceration of the perineum. On April 11th, erysipelas began to be developed in the arm as the result to irritation caused by the removal of a fragment of bone. The arm was painted with tincture of iodine and then enveloped with warm cloths. This treatment was continued until the 15th, the erysipelas continuing to spread all the time, when by advice of Professor Hamilton, who was surgeon-in-chief, the tincture of iodine was discontinued, and the arm submerged in warm water. It began to show improvement from this time, and on the 19th, all inflammation had disappeared.

Case II.—Patrick Guilfoyle, aged twenty-one, single, native of Ireland, laborer, was admitted July 14th, 1874, with a very severely lacerated and contused foot, which was amputated the same day, Syme's amputation being performed. Four days after (18th), the stump took on cellular erysipelas. It was treated with lotio plumbi et opii on sheet lint, but this seemed to aggravate the inflammation, and it rapidly extended up to the knee. Then poultices of flaxseed meal were used with slight benefit, but they were unwieldy and unclean, and could not be kept warm. This treatment was followed until the 31st, attended with lotio plumbi et opii, and the poultices, without any apparent benefit. Then the treatment was changed, the leg was enveloped with lint wet with warm water, and the whole covered with oiled silk, and in the course of twenty-four hours there was marked improvement. This was continued until August 6th, the dressings being applied twice daily, when the erysipelas had disappeared by resolution.

Case III.—John Dally, aged twenty-three, native of Ireland, laborer, came to the dispensary of this hospital July 21, 1874, with a severe laceration between the thumb and index finger of the left hand. One suture was applied in the middle of the wound and this strengthened by adhesive strips. Two days after (the 23d), he returned for redressing when it was found that the whole of the dorsum of the hand and forearm, for some distance up, was the seat of erysipelas. The whole hand and arm were wrapped in lint saturated with warm water, and then enveloped with oiled silk. In two days he returned again, the erysipelas was disappearing, and the wound suppurating. Same dressing was reapplied, and after the lapse of two days the inflammation had subsided.

Case IV.—Carl Tedesco, aged twenty-two, single, native of Germany, laborer, admitted June 10, 1874. This patient states that about eleven years ago he received a bite from a dog on the left forearm, which was cauterized on the same day, and soon the wound healed nicely. About June 10th, the patient's health being somewhat impoverished, the old wound suddenly became painful and inflamed. A hypodermic injection of morphine was given in the hand of the same side. The arm became more inflamed and soon erysipelas was well marked. The hand and forearm were submerged in warm water for an hour or two, but this being inconvenient for the patient, it was taken out and wrapped in wet lint and covered with oiled silk. After the treatment had been followed for three days the erysipelas had abated.

Case V.—Thomas Gerity, aged twenty-six, single, native of the United States, laborer, came to the dispensary on December 23, 1874, with cellular erysipelas of the forearm. The warm water dressing was applied, and in two days he returned, with some improvement and said it gave him great relief, for the pain with itching was gone. The same dressing was reapplied, and when he returned in two days all signs of erysipelas had disappeared.

### METHOD OF USING THE WARM WATER BATHS.

The baths for submersionused at the St. Francis' Hospital and recommended by Dr. Hamilton, were imported by the Franciscan Sisters, who had employed them under the direction of German surgeons during the wars between Prussia and Denmark, Prussia and Austria, and Germany and France.

Their dimensions are as follows: For the arm or hand an oblong zinc bath, 23 inches long by 8 inches wide, and 8 inches deep, with edge rolled where the arm is to enter. A movable cover extends over about two-thirds of the vessel, the remaining space being left for the arm to enter. A stop-cock is inserted at the base of the bath for the purpose of drawing off the water previous to renewal. Along the upper and outer margins of the bath are arranged small wire pins, upon which small pieces of cloth may be fastened for the purpose of sustaining the limb. Care must be taken not to allow the arm to rest against the edge of the bath so as to interfere with the circulation, and it must be adjusted at the side of the bed or chair so that the patient shall feel entirely at ease.

For the lower extremity the bath is constructed somewhat differently; a side view is very much that of an equilateral triangle resting on one angle, the opening being on the side that is uppermost. It is also provided with cover, stop-cock and rows of pins at the margings. The invention of this form of the bath is claimed by Billroth. At the suggestion of Dr. Hamilton, these baths have been much improved, especially the foot bath. The hinged legs and base have been replaced by a firm support, the stop-cock, as also in the arm bath, has been enlarged to prevent its becoming clogged by the secretions from the wound, and instead of the raised roll of metal that would press in the popliteal space, the edge has been flared outward and so covered as to present a broad surface to the limb, and admitting of a submergence of about two inches more of the leg than by the former construction.

The baths with Dr. Hamilton's improvement may be obtained of John Reynders & Co., of this city.

Independently of Dr. Hamilton, Dr. Rose has also discarded the hinged legs and base of the foot bath, and has had one made of tin with a firm circular base.

In the case of the foot bath where it is found to be comfortable to project the foot from the side of the bed, the portion of the bed upon which the body reposes is elevated by matrasses, and the bath placed upon the floor of the bedstead.

The water should be kept at a temperature to feel warm to the hand of the attendant; if when first put in the bath it is at a temperature of 100° F., it will generally need to be changed about once every four hours, of course the less the volume of water the oftener it must be changed. It would be well to use the thermometer more often than has been the custom, that the rate of falling of the temperature may be more accurately noted.

In case of a recent wound where secondary hæmorrhage is liable to occur, the limb is dressed for a few hours with warm or cold fomentations, and is left reposing in bed; but neither sutures, adhesive plasters nor bandages are applied. After all danger has passed, the warm water fomentations are continued or submersion is substituted. There is no objection to the patient's lifting the limb from the water at will, as the momentary change of position is a relief.

Submersion is only suited for the hand and arm to a few inches above the elbow, and for the foot and leg to a few inches below the knee. It has been stated that the wound should not be closed with sutures, as the swelling of tissues would cause them to tear out. This would be the rule with lacerated and contused wounds, but in some instances of incised wounds, I am informed by Dr. A. Rose, that they have been closed by sutures and immersed, and union by first intention obtained; the sutures, however, in such cases have been removed very early and before the œdema was great enough to cause them to tear out. Such results we do not look for, however, by submersion, treating simple incised wounds with fomentations. Wounds after amputations have not been submerged, as in the exposure of the ends of large arteries there is a possibility of secondary hæmorrhage. Fomentations are here employed.

After submersion for about fourteen days, warm water fomentations are generally substituted, and continued until the wound heals. In a few cases, patients have been unable to keep the limb in the bath for more than six or eight days, on account of the pain produced. The only remedy for this is to remove the limb from the bath and use fomentations.

In using fomentations, the limb is wrapped in several folds of sheet lint or soft old muslin, saturated with warm water and covered with some material that will prevent rapid evaporation, such as oiled silk or sheet rubber. The water should be renewed about every four hours, or often enough to keep the lint moist and warm.

Summary.—We have seen how, from the earliest ages which we have received any records of medicine, simple water has been used, both externally and internally, to allay inflammatory action ; how Hippocrates, in the fourth century B. C., used warm water extensively ; Celsus, about the first century A. D., recommended cold water for slight injuries, but if "inflammation is active," warm water is to be employed ; and Galen, in the second century A. D. used warm water in wounds.

In the seven centuries constituting the "middle ages," water was used as a therapeutic agent, but subjective to superstitious ceremonies.

In the sixteenth century, Ambrose Paré recommended cold water. Lamorier, in the eighteenth century (1732), reports the use of warm water submersion in the treatment of an old ulcer on the ankle, and in two cases of suppurating wounds of the arm and hand. In the same century it was employed in Germany, having Theden as its special advocate.

In the present century (1808), we have seen with what satisfactory results five hundred soldiers suffering from gun-shot injuries, mostly in the feet, were treated with water-dressings by Treille.

Josse, of Amiens, about 1830, instituted irrigation, which was introduced in the Hotel Dieu, at Paris, by Breschet in 1834.

Sanson (1831) treated many cases of compound fracture with water, and by its use had prevented traumatic fever.

Twenty-five years ago, Amussat was the especial advocate of water treatment in surgery. He preferred a temperature of 64° to 68° F.

From that time down it has been used in a desultory manner by the French, but more generally by the Germans. It was employed very extensively in the army in the wars of Germany with Denmark, Prussia and France, a temperature being employed that should be most agreeable to the patient.

The present method of using water in Germany is mainly by submersion, according to the principles and method described by Dr. Max Schede, from whose paper I have so largely drawn in the foregoing pages.

The use of hot water in surgery having been suggested, as far as I have been able to learn, by Prof. Frank H. Hamilton, about a year ago.

From the experience given, I think we have ample testimony to the efficacy of water, and especially warm and hot water, in the treatment of surgical affections, such as lacerated and contused wounds, ulcers, sprains, compound fractures, after operations, etc.

As to cold water, 68° F. and below, it has been used to stop parenchymatous hæmorrhage and to reduce inflammation, both acute and chronic. Water at this temperature is a good hæmostatic, but, as Dr. Schede observes, it causes the patient great suffering. To be effective in the reduction of inflammation, it must be long continued, and reaction must be carefully guarded against ; at 32° F. gangrene may be induced by the prolonged anæmia, caused by the contraction of the smaller arteries and the capillaries.

At a temperature of 77° to 86 F. tepid water, we have seen how the most severe of recent wounds may be successfully treated ; inflammation prevented and gangrene restrained to those tissues only which were devitalized at the time of the accident, members being saved to the patient that, by other than water treatment, must have in all probability been amputated. This is demonstrated very clearly in the cases of injury of the hand recorded by Dr. Shede. But wounds in which suppuration is already established, in which pus has burrowed, forming sinuses, cannot be treated as well at this temperature as at a higher, as the coagulated and retained pus undergoes decomposition and renders the patient liable to septicæmia, which, as far as I have observed, has not occurred with water at a higher temperature.

As hot water, 100° F., or above, however, I think we have a temperature suited to a greater variation in the condition of wounds than either of the temperatures before mentioned, or than even the temperature of 90° to 98° F., warm water. Hot water is an excellent hæmostatic, and is especially useful after an operation to stop the oozing from the cut surfaces of both flesh and bone. In recent injuries it prevents inflammation and traumatic fever. When erysipelatous inflammation has occurred, it has disappeared soon after the commencement of the hot-water treatment. Hot water is, however, especially useful in that class of cases where suppuration is already established, and where gangrene has occurred; in the latter condition it speedily restores the circulation of the dying parts, preventing the extension of gangrene, and causes a rapid separation of the dead mass; in the former it acts as a stimulant to the tissues, encouraging granulations which become *decided*, *bright* and *healthy* under its action.

Of the different methods of applying the water, submersion with warm or hot water has been found to be the best, where the wound is in a location to admit of it, as in the forearm, hand, leg or foot. If the wound is upon some other part of the body, fomentations are to be preferred.

There are many other conditions in which hot water is an excellent therapeutic agent. Bed sores have been cured by its application. Hot-water sitz-baths, I have found an excellent remedy in dysmenorrhœa; the patient to take a sitz-bath nightly for two weeks before the menstrual period; a repetition of the treatment before a second menstrual period, has caused the flow to take place without any intimation of it to the patient, the pain accompanying the flow during the first two or three months being entirely absent.

In treatment of inflammatory affections of the uterus, Dr. Thomas A. Emmet employs internal water douches at as high a temperature as the patient can bear.

In acute articular rheumatism, it is always worthy of a trial, not only to relieve the pain, but to improve the circulation in the joints and soften the tissues. Hueter says that "in cases of acute articular rheumatism, polyarthritis synovialis acuta, the hot-water bath is to be recommended, and in gout, polypanarthritis, I know of nothing better."

The history of a case of acute articular rheumatism, with heart lesion producing regurgitant murmur, as obtained from the family of the patient, is as follows:

A girl, five years of age, had been under treatment for three months. She had been confined to the bed during this time and was rapidly wasting away with the great pain that she suffered. The legs had become flexed on the thighs, and the thighs to a right angle with the body, and both limbs so abducted as to lie flat upon the bed, bed-sores having formed upon both external malleoli.

All internal remedies having been laid aside, and no tendons cut, hot-water treatment was instituted. Suffering too much to be removed to a bath at first, flannels were saturated with boiling water, wrung out and applied for an hour or two each day for a week; *hot baths* were then commenced, the patient being carried in a sheet and placed in water at a temperature of 105° F. After the first bath she slept for two hours and awoke refreshed and without pain—the first time in two months. After using the baths for two weeks, with manipulations of the joints, the patient was removed from the bed and placed in a reclining chair. The baths were continued for a month longer; she now remained free from pain most of the time, and when pain was experienced, it was promptly relieved by *hot*-water fomentation. The patient was now removed to the country, where she remained about two months, the manipulations and fomentations being continued. She was restored to the use of her feet and the use of crutches in about a month longer. Eight or ten weeks later, the crutches were taken from her, and she gradually obtained full control of her limbs.

The following case of atony of the bladder and spasmodic stricture of the urethra, under my own observation, was finally cured by the use of hot-water sitz-baths:

The patient, a domestic, aged twenty-two years, had slipped at the top of a flight of six steps, struck upon the middle of the posterior surface of the left thigh, and slid to the bottom. A severe contusion was produced, but the skin was not broken; experienced some shock, but soon recovered and went about her work. The next day she felt sick, her hands and feet were cold, and she went to bed. On the third day, having passed no water since the injury was received, about one and one-half pints were drawn with a catheter.

Urine was not passed naturally until the seventh day, when some pain was experienced, the catheter being used in the mean twice a day. During this time she had felt sore and lame, as the result of the injury and the continuance of her house duties. There was also some loss of sensation in the injured limb. On the fourteenth day, acetate of potash was prescribed, somewhat increasing the quantity of urine and making it alkaline. Otherwise, from the eleventh to the eighteenth days, a small quantity was passed naturally once a day and the catheter used once a day.

As from this experience it became apparent that this treatment would have to be continued indefinitely, it was decided to make a change, and a hot sitz-bath at as high a temperature as could be was directed to be used upon retiring, the result of which was to induce natural urination, free from pain, about two hours after its employment. The sitz-baths were used for about ten days, by which time the urinary function was completely restored. I conclude that if the hot sitz-baths had been used at first, catheterism would have been unnecessary.

It is evident from the above experience that catheterism, so much objected to, especially by females, might in many cases be entirely avoided by the timely use of the hot sitz-bath.

The therapeutic uses of warm water, 70° to 98° F., and hot water, 98° to 110° F., could undoubtedly be much more extensively dilated upon; but as I have not intended to write an exhaustive treatise on the subject, but simply to present in a compact form some of the facts in relation thereto, I will rest here.

## Figures and Tables

**Figure f1:**
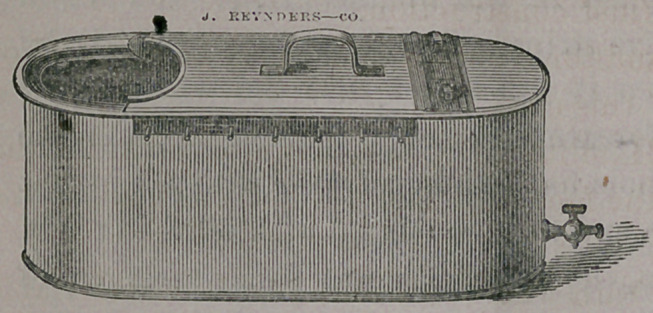


**Figure f2:**